# Ninjurin1 inhibits colitis-mediated colon cancer development and growth by suppression of macrophage infiltration through repression of FAK signaling

**DOI:** 10.18632/oncotarget.9020

**Published:** 2016-04-26

**Authors:** Jong Kyu Woo, Yeong-Su Jang, Ju-Hee Kang, Jong-Ik Hwang, Je Kyung Seong, Sang-Jin Lee, Sejin Jeon, Goo Taeg Oh, Ho-Young Lee, Seung Hyun Oh

**Affiliations:** ^1^ College of Pharmacy, Gachon University, Incheon, Republic of Korea; ^2^ College of Veterinary Medicine, Seoul National University, Seoul, Republic of Korea; ^3^ National Cancer Center, Goyang-si, Gyeonggi-do, Republic of Korea; ^4^ Graduate School of Medicine, Korea University, Seoul, Republic of Korea; ^5^ Department of Life sciences, Ewha Womans University, Seoul, Republic of Korea; ^6^ College of Pharmacy, Seoul National University, Seoul, Republic of Korea

**Keywords:** macrophage, ninjurin1, colon cancer, colitis-associated colon cancer model (CAC), focal adhesion kinase (FAK)

## Abstract

Macrophage infiltration promotes tumorigenesis. However, the macrophage infiltration regulatory molecules have not been fully determined. Nerve injury-induced protein 1 (ninjurin1) is a homophilic cell surface adhesion molecule that plays an important role in cell migration and attachment. Although ninjurin1 is believed to play a role in several malignancies, it is unclear whether ninjurin1 expression contributes to cancer progression. We used transgenic mice (tg mice) that overexpressed ninjurin1 on macrophages. We subjected ninjurin1 tg mice to a well-known mouse model of colitis-associated colon cancer in which animals are treated with azoxymethane (AOM) and dextran sulfate sodium (DSS). After AOM and DSS treatment, ninjurin1 tg mice developed fewer and smaller tumors compared with wild-type (wt) mice. Ninjurin1 tg mice also showed reduced infiltration of macrophages and suppressed angiogenesis in the tumor mass. We therefore explored whether ninjurin1 decreases macrophage migration into the tumor sites. After adoptive transfer to tumor-bearing recipients, wild type and ninjurin1 tg mice's peritoneal macrophages were freshly isolated and labeled with carboxyfluorescein succinimidyl ester (CFSE). As expected, compared with that of wt type macrophages, tumor infiltration of ninjurin1-overexpressing macrophages was significantly decreased. We also found that ninjurin1 overexpression suppressed FAK activity. In addition, knockdown of ninjurin1 enhanced FAK activity and migration activity of RAW264.7 cells. Ninjurin1 overexpression on macrophage inhibits tumor growth by suppression of macrophage infiltration through repression of FAK signaling. Ninjurin1 is a key regulator molecule for macrophage migration and Tumor-associated macrophages (TAM) mediated tumorigenesis *in vivo*.

## INTRODUCTION

Macrophages are involved in a variety of processes including pathogen destruction, inflammation, tissue repair and remodeling [1]. Mononuclear phagocytes are an essential element in the orchestration and expression of innate immunity and adaptive immune responses. These cells play a central role in inflammation and host defense [2]. They have a highly plastic phenotype and their functional polarization is determined by cytokines and factors found within local microenvironments [3]. Diversity and plasticity have long been known to be characteristics of monocyte–macrophage lineage cells.

Tumor-associated macrophages (TAMs) represent a substantial fraction of the growing tumor mass and are associated with poor prognosis in several human cancers [4]. TAMs exist in two different polarization states. In response to various signals, macrophages may undergo classical M1 activation or alternative M2 activation [5, 6]. M2-like TAMs are responsible for many tumor-promoting activities during tumor initiation, progression, angiogenesis and metastasis [7]. M2-like TAMs play a major role in suppressing the anti-tumor responses of dendritic cells (DCs), cytotoxic T lymphocytes (CTLs), and natural killer (NK) cells [1]. Angiogenesis, the formation of new blood vessels, is also required for tumor expansion, progression, and metastasis [8]. TAM infiltration affects angiogenesis by influencing the production of angiogenic cytokines and growth factors including interleukins, tumor necrosis factor-α (TNF-α), IFN-γ, basic fibroblast growth factor (bFGF), thymidine phosphorylase (TP), urokinase-type plasminogen activator (uPA) and vascular endothelial growth factor (VEGF) [9]. Therefore, modulation of TAMs may be critical in nascent vessel formation in tumors, and could be targeted to inhibit tumor growth.

Ninjurin1 (nerve injury-induced protein 1) a homophilic adhesion molecule and cell surface protein, is highly induced following nerve injury in dorsal root ganglion (DRG) neurons and Schwann cells [10]. Like other cell adhesion molecules, ninjurin1 plays diverse roles in pathological conditions and is involved in many human diseases like Spinal cord injury [10] and leprosy [11]. Ninjurin1 is also believed to play an important role in hepatocellular carcinoma [12] and B-cell leukemia [13]. However, the biological significance of ninjurin1 expression in macrophages remains controversial.

TAMs are considered attractive targets for anti-tumor intervention [14]. A previous study showed that the pharmacologic depletion of macrophages in different mouse tumor models significantly reduced tumor angiogenesis and progression, suggesting that macrophages are critical components in the tumor microenvironment for tumor progression [15]. Indeed, blocking the functions of TAMs inhibits tumorigenesis. Accumulating evidence has shown that TAMs enhance tumor progression to malignancy in the majority of cases [16, 17]. There has been growing interest in studying the regulating molecules of macrophage infiltration in solid tumors. Ninjurin1 proteins were up-regulated in myeloid cells associated with experimental allergic encephalomyelitis and active multiple sclerosis, and this subsequently modulated the infiltration of inflammatory myeloid cells into the central nerve system [18]. These reports let us postulate that ninjurin1 plays a role in macrophage migration and infiltration during tumorigenesis.

In the present study, we explored whether ninjurin1 overexpression suppresses macrophage migration or invasion in subcutaneous lung cancer xenograft model. In addition, we investigated whether ninjurin1 suppresses FAK signaling pathway in macrophages. Based on these data, we suggest that ninjurin1 proteins are able to repress tumor growth when overexpressed on macrophages of transgenic mice.

## RESULTS

### Ninjurin1 overexpression on macrophages significantly decreases colitis-associated colon cancer incidence

**Figure 1:**
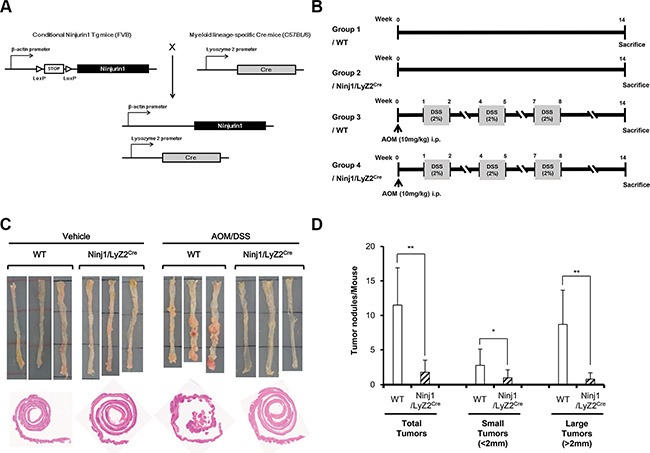
Response of ninjurin1 overexpression on macrophage in DSS/AOM-mediated colon cancer model (**A**) Scheme of the generation of mouse strain that expresses conditional ninjurin1. The STOP cassette, which prevents expression of functional ninjurin1, is removed by crossing the ninjurin1 strain to a *Lyz2*-Cre myeloid cell lineage-specific Cre-expressing mouse strain. (**B**) Protocol for AOM/DSS-induced colon carcinogenesis. Wt and ninjurin1 tg littermates were intraperitoneally injected with AOM (10 mg/kg) then subjected to 3 cycles of DSS treatment (1 cycle represents 1 week of 2% DSS treatment followed by 2 weeks of water treatment). After 11 weeks of AOM/DSS exposure, the mice were killed. Colons were examined for counting or measuring tumors and then fixed and paraffin embedded. (**C**) Representative colon pictures at the end of the experiment. (**D**) Tumor nodules and tumor numbers/mouse in WT and ninjurin1 tg mice. Size distribution of tumors is also shown. Differences were evaluated using an unpaired two-tailedStudent's *t*-test. (Error bars denote the standard deviation [SD]) (*p < 0.05 and **p < 0.01).

Ninjurin1 expression is known to be associated with recruitment of specific immune cell populations in inflammatory sites [18]. To identify the novel function of ninjurin1 in tumorigenesis, we first addressed whether overexpression of ninjurin1 on macrophages altered the susceptibility to develop CAC. Wt and ninjurin1 tg mice were treated with AOM/DSS as described in the materials and methods section (Figure 1A and 1B). AOM induces tumors in the distal colon of rodents and is commonly used to elicit colon cancer in experimental animals [19]. We found a striking difference between wt and ninjurin1 tg mice with respect to development of polyps and dysplasia. All wt mice grossly showed multiple polypoid lesions, but no such visible lesions were seen in ninjurin1 tg mice (Figure 1C). The number of colonic tumors was decreased by ~80% in ninjurin1 tg mice. Ninjurin1 tg mice also showed reduced tumor multiplicity compared with wt mice (11.5 ± 5.40 vs. 1.8 ± 1.75, *p* < 0.01) (Figure 1D). Furthermore, the numbers of tumors larger than 2 mm were significantly less in ninjurin1 tg mice than in wt mice (8.7 ± 4.95 vs. 0.8 ± 0.92, *p* < 0.01) (Figure 1D). These results indicate that ninjurin1 overexpression suppresses the growth of colorectal tumors in a mouse model of CAC.

### Macrophage infiltration is decreased in ninjurin1 tg mice

**Figure 2:**
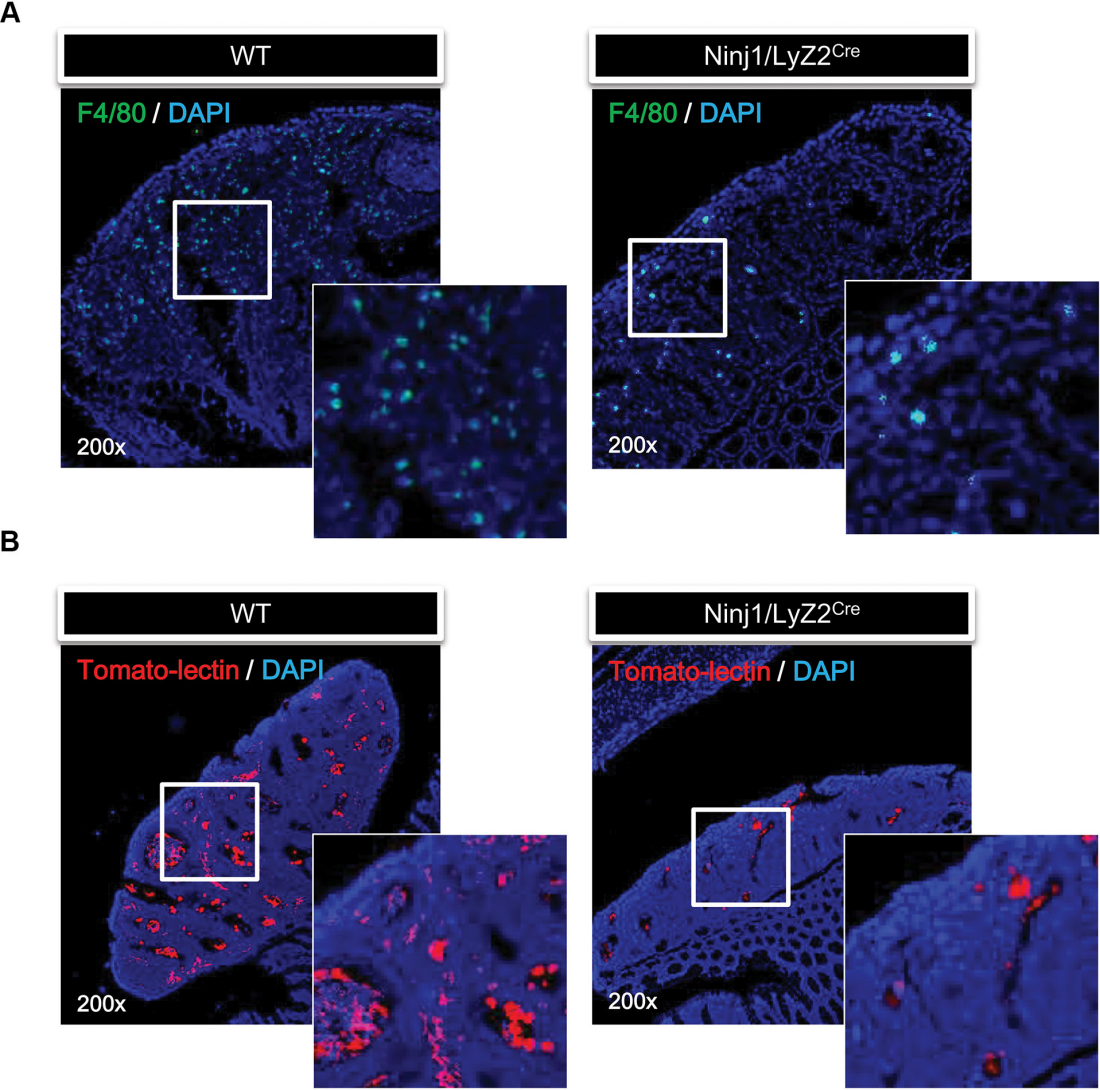
Overexpression of ninjurin 1 on macrophage results in the inhibition of tumor infiltration and angiogenesis (**A**) Immunofluorescence staining of F4/80 was performed to determine macrophage infiltration. Representative immunofluorescence pictures of colonic sections taken from wt and ninjurin1 tg mouse and stained with FITC conjugated F4/80 antibody. (**B**) Fluorescence staining with texas-red tomato-lectin in AOM/DSS induced colon tumors. AOM/DSS induced colon tumors were harvested from wt and ninjurin1 tg mice 6 h post intra-cardiac injection of texas-red tomato-lectin. The red fluorescent staining indicates endothelial cells. DAPI was used as a nuclear counterstain.

We examined whether macrophages infiltration and angiogenesis are associated with expression of ninjurin1. Macrophage infiltration is a useful diagnostic marker for the progression of cancers. Macrophages are critical for the growth and angiogenesis of tumors [20, 21]. To examine whether macrophages were reduced in tumors of CAC model mice, some animals were sacrificed and tumors were excised for histology. Microscopic examination of immunofluorescence revealed tumor infiltration by macrophages in wt mice, but little infiltration in ninjurin1 tg mice (Figure 2A). These differences were associated with ninjurin1 expression on macrophages. To investigate whether ninjurin1 reduces tumor angiogenesis *in vivo*, we stained the tumor with texas-red conjugated tomato-lectin. Consistent with macrophage infiltration, ninjurin1 tg mice had remarkably fewer blood vessels compared with wt counterparts (Figure 2B). These results show that ninjurin1 overexpression on macrophage can inhibit macrophages infiltration into tumors and suppress angiogenesis.

### Phenotype of macrophages from ninjurin1 tg mice

**Figure 3:**
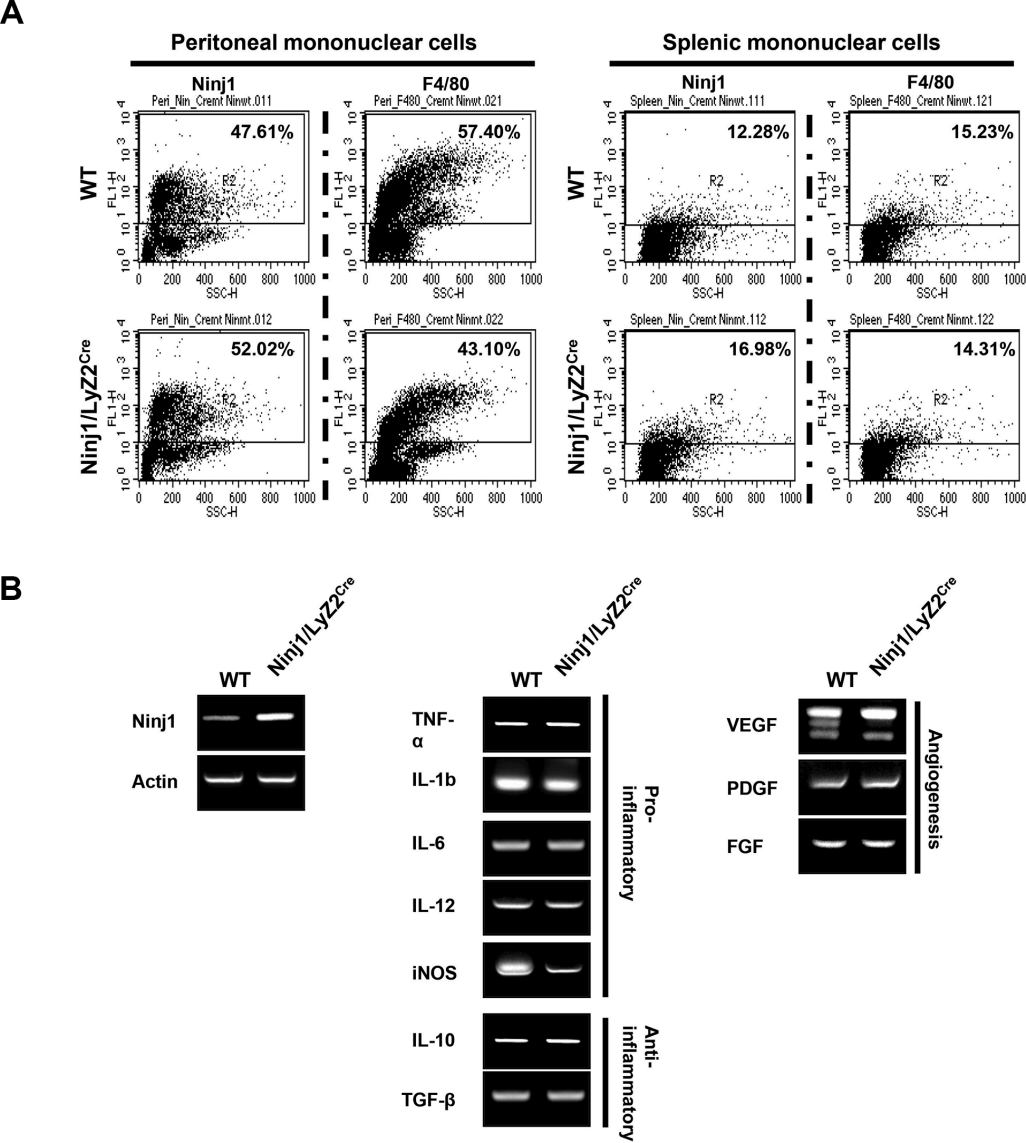
Characteristics of ninjurin 1-overexpressing macrophages (**A**) Flow cytometry analysis of peritoneal and splenic macrophages isolated from wt and ninjurin1 tg mice. (**B**) Multiple PCR analyses of inflammatory related cytokines and angiogenesis related growth factors in peritoneal macrophages.

We tested whether ninjurin1 affects the characteristics of macrophages *in vivo*. Peritoneal resident cells and splenic mononuclear cells were collected and stained with macrophage marker and sheep anti-ninjurin1 antibodies. Flow cytometry analysis of cells isolated from wt mice and ninjurin1 tg mice confirmed that ninjurin1 tg mice shown more F4/80 or ninjurin1 positive cell populations. Ninjurin1 overexpression caused a rapid decrease in the number of peritoneal resident macrophages, while splenic macrophages were the same as in wt. However, surface ninjurin1 expression was increased on peritoneal resident cells and splenic mononuclear cells compared with wt mice counterparts (Figure 3A). Next, we examined the expression profiles of cytokines and growth factors by macrophages secretion for regulation of tumor microenvironments (Figure 3B). Peritoneal resident macrophages were isolated from wt mice and ninjurin1 tg mice and subjected to semi-quantitative RT-PCR. Macrophages from ninjurin1 tg mice showed higher expression of ninjurin1 tg than wt macrophages. Pro-inflammatory genes (*TNF-α, IL-1β, IL-6, IL-12, and iNOS)* were similarly expressed in macrophages from wt and ninjurin1 tg mice (Figure 3B). Also, mRNA levels of the anti-inflammatory cytokines, *IL-10* and *TGF-*β were similarly expressed in both macrophages (Figure 3B). Furthermore, wt macrophages and ninjurin1 tg macrophages showed similar expression of *VEGF, PDGF*, and *FGF2* mRNA. Taken together, these data suggest that ninjurin1 overexpression on macrophages does not repress tumor growth via anti-inflammatory or anti-angiogenic activity but via suppression of tumor infiltration capacity of macrophages. During tumor progression, circulating monocytes and macrophages are actively recruited into tumors. Infiltrated macrophages are polarized to TAM. Experimental and animal studies have reported that TAM can provide a favorable microenvironment to promote tumor development and progression. The ninjurin1 protein is a crucial regulator of macrophage adhesion and migration. We emphasize here a strong inverse correlation between macrophage infiltration inhibition by ninjurin1 and tumor angiogenesis. Consistent with our result, some tumor and stroma-released cytokines and chemokines facilitate the recruitment of macrophages to tumor tissues. Therefore, inhibiting macrophage recruitment by modulating the adhesion or migration has become a promising approach for tumor therapy.

### Ninjurin1 overexpression represses infiltration of macrophages to tumors

**Figure 4:**
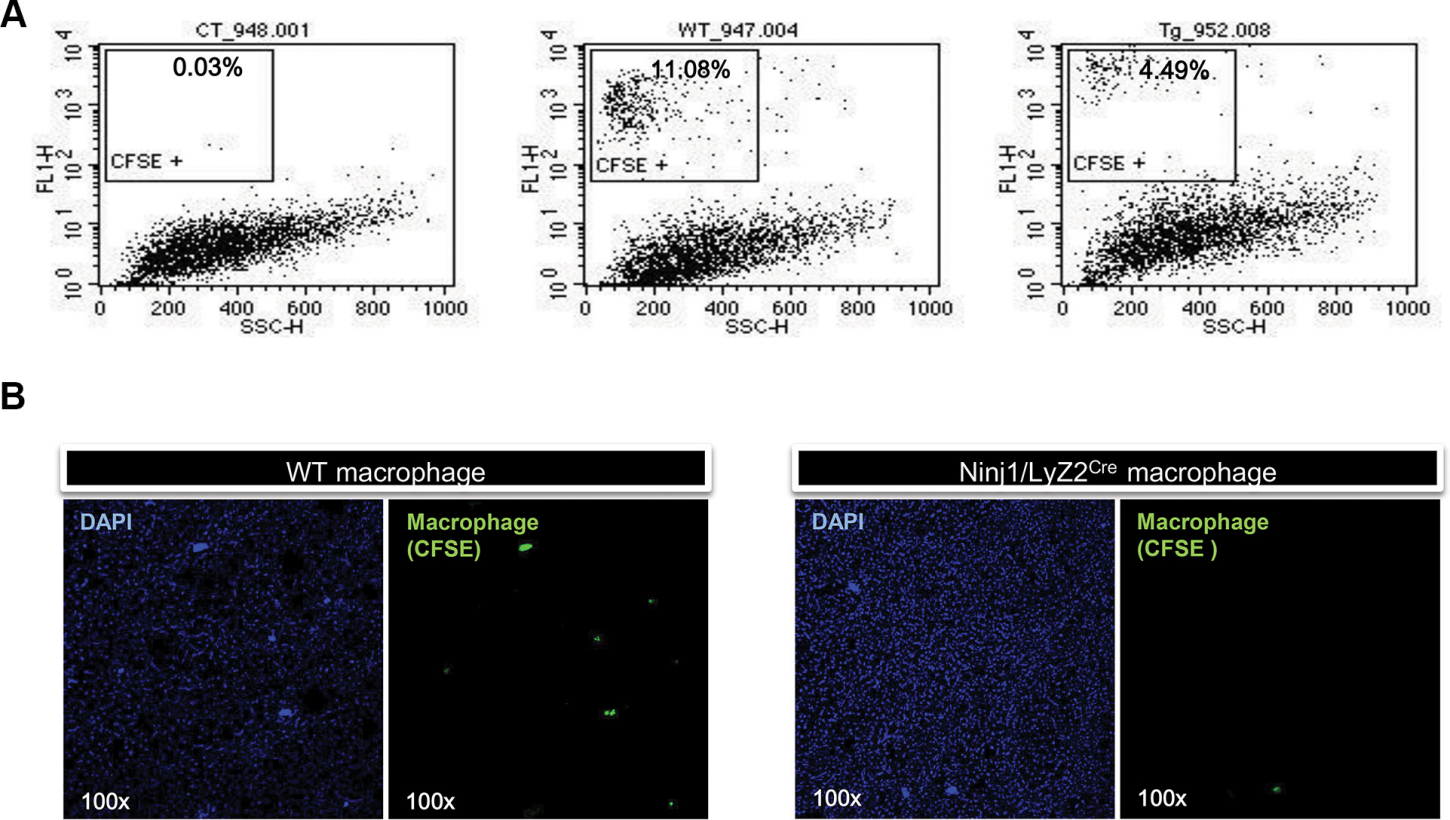
Ninjurin 1 overexpression on macrophages reduced the number of tumor infiltrated macrophages (**A**) Representative histogram with percentages of CSFE positive cells. (**B**) CFSE labeled macrophages were observed in xenograft tumors collected from recipient mice.

To determine whether ninjurin1 has a cell autonomous role in macrophages during tumor development, adoptive transfer of macrophages to A549 tumor transplanted NOD/SCID mice was performed. Macrophages were isolated from peritoneum of wt and ninjurin1 tg mice and were labeled with CFSE fluorescent dye. CFSE-labeled macrophages were injected into the left ventricle of the heart of recipients. After 24 h, tumor infiltrated CFSE-labeled cells were analyzed by flow cytometry. Flow cytometry analysis demonstrated that CFSE positive populations were highly present in wt mice compared with ninjurin1 tg mice (Figure 4A). To directly visualize infiltrated macrophages, we performed immunofluorescence analysis on tumor sections of NOD/SCID mice. Consistent with results obtained from FACS analysis, macrophage numbers from wt mice were much higher than from ninjurin1 tg mice (Figure 4B). Furthermore, we adoptively transferred macrophages from ninjurin1 knock-out mice into A549 tumor transplanted NOD/SCID mice. 24 hours after adoptive transfer, we determined the ratio of infiltrated CFSE-positive monocytes (Supplementary Figure 1D). Flow cytometry analysis proved that CFSE positive populations were highly present in ninjurin1 knock-out mice compared with wt mice. Thus, our results demonstrate that cell-autonomous expression of ninjurin1 on macrophage inhibits macrophages infiltration to tumors.

### Ninjurin1 overexpression suppresses macrophages migration and invasion through repression of FAK signaling

**Figure 5:**
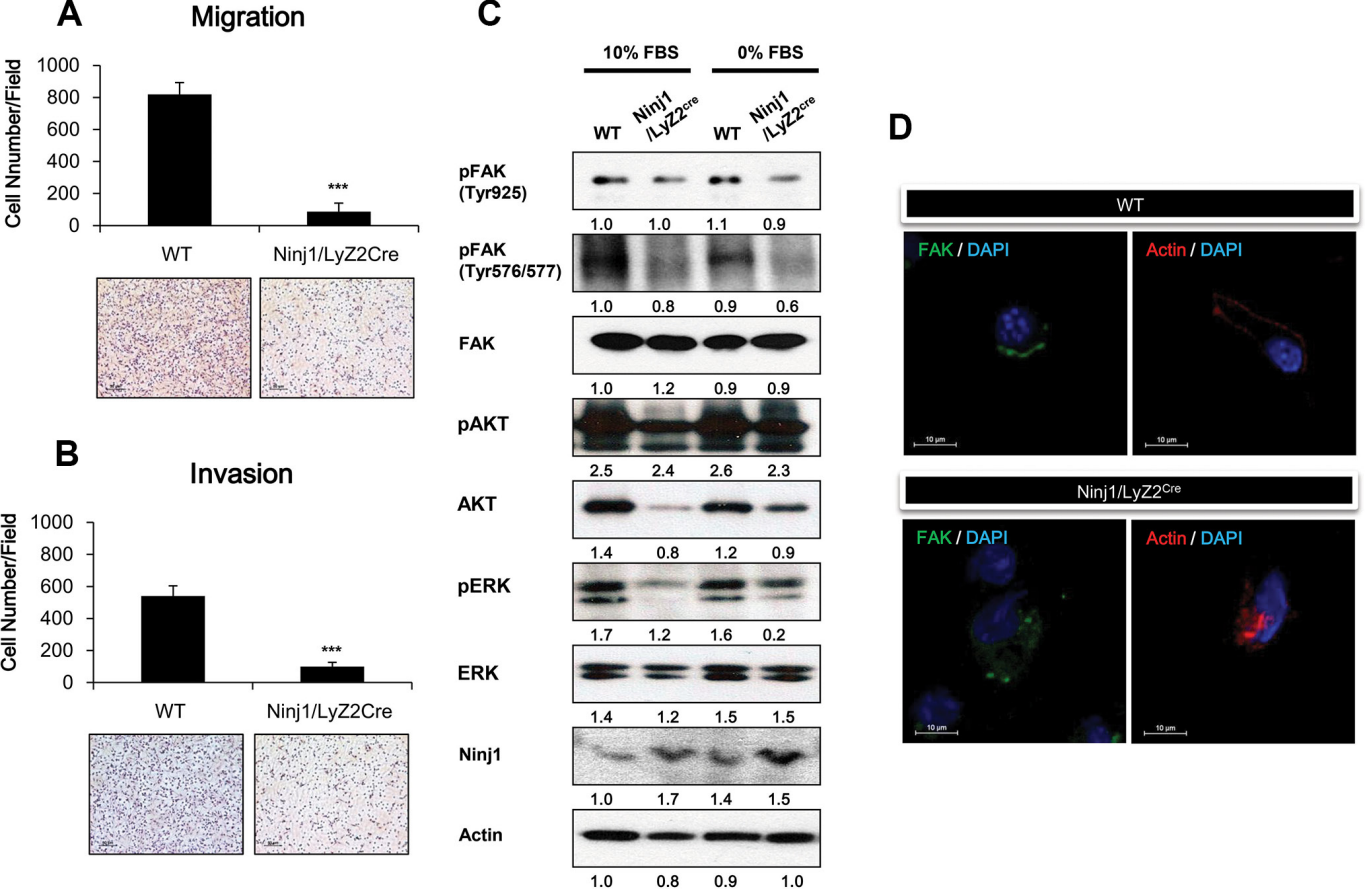
Ninjurin 1 overexpression on macrophage decreases macrophage migration and invasion through inhibition of FAK activity Ninjurin1 overexpression suppresses cell migration (**A**) and invasion (**B**). Migrated and invasive macrophages on outer surface of the upper chambers were stained with H&E. A representative image of Transwell migration and invasion assays is shown. The penetrated cells were counted in three independent experiments. Differences were evaluated using an unpaired two-tailed Student's *t*-test. (Error bars denote the standard deviation [SD]) (*p < 0.05 and **p < 0.01). (**C**) FAK, phosphorylated FAK, phosphorylated AKT and phosphorylated ERK levels were evaluated by western blot analysis in cultured macrophages with 10% FBS or 0% FBS. (**D**) The fluorescence images show FAK (green) and actin (Red) Immunostaining.

To measure the effect of ninjurin1 on macrophage migration and invasion, transwell assays were performed. In the migration assay, we found that macrophages isolated from ninjurin1 tg mice exhibited 86.7 ± 52.9 cells/field and macrophages from wt mice exhibited 819 ± 73.6 cells/field. In the invasion assay, we found that macrophages isolated from ninjurin1 tg mice exhibited 9.56 ± 2.43 cells/field and macrophages from wt mice exhibited 29.69 ± 7.34 cells/field (Figure 5A and 5B). Ninjurin1 knock-out mice were also used for the experiment. Ninjurin1 knock-out macrophages (1528.5 ± 127.6) migrated faster than wt macrophages (619 ± 39.2). It should be noted that ninjurin1 knock-out macrophages were not especially invasive compared with wt macrophages (ninjurin1 knock-out 220 ± 33.7 vs wt 200.5 ± 33.7) (Supplementary Figure 1A and 1B). In altogether, these results indicate that ninjurin1 directly reduced the migration and invasion of macrophages. Focal adhesion kinase (FAK) is a non-receptor kinase that plays an important role in signal transduction and is a key regulator of survival, proliferation, migration and invasion [22, 23]. Phosphorylation of FAK leads to cell migration and motility by eliciting focal adhesion formation [24]. We performed western blotting to investigate whether FAK signaling pathways were altered by ninjurin1 overexpression in macrophages. We measured FAK activity by monitoring the phosphorylation of FAK^Y925^ and FAK^Y576/577^, respectively. Macrophages isolated from wt and ninjurin1 tg mice peritoneal were cultured in the presence or absence of FBS, and then the levels of FAK^Y925^, FAK^Y576/577^ and phosphorylated AKT and phosphorylated ERK were analyzed. As shown in Figure 5C, FAK activation was significantly suppressed by ninjurin1 overexpression. Phosphorylation of FAK downstream kinases, AKT and ERK was also decreased by ninjurin1 overexpression. To confirm the transgenic macrophages data, we cultured knock-out macrophages in the presence or absence of FBS, and measured levels of FAK^Y925^, FAK^Y576/577^ phosphorylated AKT and phosphorylated ERK (Supplementary Figure 1C). FAK activation was significantly induced by ninjurin1 deprivation. In addition, to investigate the dynamic of FAK and actin filaments in migrating cells, we stained macrophages with anti-FAK or anti-actin. Figure 5D shows the fluorescence images of FAK and actin at the cell periphery. Immunofluorescence analysis revealed the loss of FAK and actin clusters at the cell leading edge in ninjurin1 tg macrophages but not in wt macrophages. These immunofluorescence results are consistent with the migration and invasion assays, showing that ninjurin1 overexpression suppresses cell mobility. Collectively, these data demonstrated that macrophage migration and invasion are suppressed by ninjurin1 through suppression of FAK signaling pathways.

### Ninjurin1 knock-down suppresses migration and invasion in RAW264.7 cells

**Figure 6:**
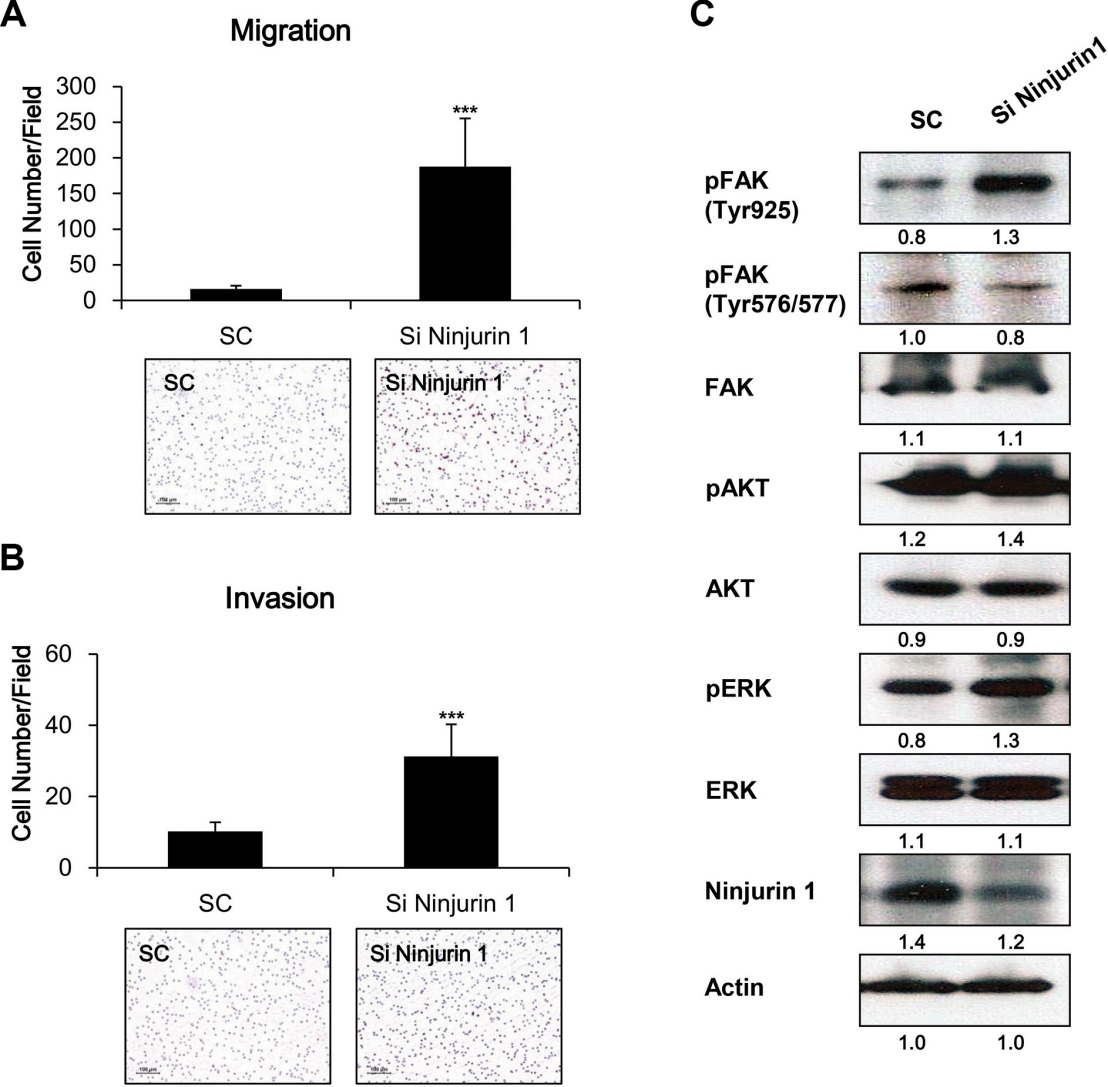
Depletion of ninjurin 1 from RAW264.7 cells increased macrophage migration and invasion, and enhanced FAK activity RAW264.7 cells were transfected with control siRNA (SC) or ninjurin1 siRNA (Si Ninjurin1) and seeded in the upper layer of transwell and the cells were incubated for 12 h. Migration (**A**) and invasion (**B**) of siRNA transfected RAW264.7 cells were detected using H&E staining. Differences were evaluated using an unpaired two-tailed Student's *t*-test. (Error bars denote the standard deviation [SD]) (*p < 0.05 and **p < 0.01). (**C**) Expression of FAK, phosphorylated FAK, phosphorylated AKT and phosphorylated ERK in ninjurin1 siRNA- transfected RAW264.7 cells were detected by western blotting.

To further evaluate the influence of Ninjurin1 on cell migration and invasion, RAW264.7 murine macrophage cells were transfected with siRNA ninjurin1 to knockdown endogenous ninjurin1. We observed increased migration (Figure 6A) and invasion (Figure 6B) in ninjurin1 siRNA-transfected RAW264.7 cells. On the other hand, we examined whether FAK activity is altered by ninjurin1 knockdown. As shown in Figure 6C, FAK^Y925^ and FAK^Y576/577^ were down-regulated by ninjurin1 siRNA transfection. Together, these data indicate that ninjurin1 knockdown increases migration and invasion of RAW264.7 murine macrophage cells through activation of FAK signaling pathways

## DISCUSSION

Macrophages remain focus of scientific interest because of their integrative functions in diverse physiological and pathophysiological processes, including tumor development. Our results demonstrate that ninjurin1 overexpression on macrophages decreases mobility of macrophages and suppress tumor growth. We illustrate that ninjurin1 can directly regulate migration and invasion of macrophages through suppression of FAK activation. Ninjurin1 protein is constitutively expressed in monocyte lineages and is involved in neuron-glia interactions, promoting axonal sprouting and nerve repair. In addition, ninjurin1 overexpression enhances adhesion between BV2 cells (murine monocytes lineage microglia) and endothelial cells [25]. We demonstrate that, ninjurin1 overexpression suppresses macrophage infiltration and tumor growth.

In the present study, we established the *Lyz2* Cre/LoxP-Stop-LoxP-ninjurin1 mouse, which is characterized by restricted expression of ninjurin1 in the myeloid cell lineage. The myeloid cell-specific expression of ninjurin1 is mediated by expression of Cre recombinase under control of the *Lyz2* promoter; *Lyz2*-driven Cre recombinase excises a loxP-flanked transcriptional STOP cassette in front of the ninjurin1 open reading frame, thus allowing expression of ninjurin1 in the myeloid cell lineage. Initially, ninjurin1 tg mice were produced to clarify the role of ninjurin1 in the process of colitis-induced colon carcinogenesis. AOM treatment followed by repeated cycles of DSS resulted in chronic inflammation and the development of colon tumors in the treated mice. Ninjurin1 overexpression suppressed colon cancer development and reduced tumor size. In addition, macrophage-specific ninjurin1 overexpression reduced the number of infiltrated macrophages and angiogenesis in the tumors. This observation could be the result of decreased macrophage migration.

Since macrophages are known tumor growth and angiogenesis regulators [9, 15], it is surprising to find that infiltrated macrophage numbers were critically suppressed by ninjurin1 overexpression on macrophages. One basic question is to clarify the mechanism by which ninjurin1 derived inflammatory cytokines regulate tumor growth. However, the reduced tumor burden in ninjurin1 tg mice did not correlate with expression of inflammatory cytokines and angiogenesis related growth factors. Taken together, our data favor a model of ninjurin1 on macrophages suppressing tumor growth via reduction of tumor infiltration by macrophages. These results were confirmed by functional studies in macrophage adoptive transfer that also showed less ninjurin1-overexpressing macrophage infiltration than wt macrophage. This observation could be the result of decreased migration of monocytes from circulating blood into tumors.

Focal adhesion kinase (FAK) is a well-known cell mobility regulation kinase. FAK regulates cell-matrix interaction and integrin signaling resulting in cell spreading, migration and survival [26]. During migration, FAK coordinates lamellipodial formation and the turnover/disassembly of focal adhesion [27]. In this study, we demonstrate for the first time that FAK activation is suppressed by ninjurin1 overexpression in macrophages. Based on these findings, we propose that ninjurin1 is constitutively expressed in macrophages and might play a housekeeping role in macrophage physiology.

It is important to know how FAK is suppressed by ninjurin1 overexpression. Classically, FAK is a tyrosine kinase that is a central molecule in integrin-mediated signal transduction, and it is involved in cellular motility and protection against cell death [28, 29]. A very interesting clue is that ninjurin1 is a membrane protein that directly interacts with itself on the cell membrane. Under these circumstances, the overexpression of membrane ninjurin1 molecules can inhibit integrin clustering. Considering the effect of ninjurin1 on FAK inhibition, the inhibition of FAK phosphorylation in macrophages may proceed primarily through the inhibition of the clustering of outer membrane proteins. Thus, we presume that direct or indirect inhibition of the association of integrin or other outer membrane molecules might suppress the macrophages migration. Additionally, tumor infiltration of macrophages could be efficiently targeted, at least partially, depending on ninjurin1-mediated FAK inhibition. Results from this study confirm that ninjurin1 is an important regulator of FAK activation during macrophage tumor infiltration. However, further studies are needed to clarify the underlying mechanisms.

Thus, a regulated balance between cytoskeletal assembly and disassembly must be maintained to ensure cell movement. Loss of tyrosine phosphorylation of FAK is associated with disruption of focal adhesions and acquisition of a motile phenotype [30]. FAK may also promote cell migration by influencing the remodeling of the actin cytoskeleton [31]. Our data revealed that ninjurin1 overexpression suppresses FAK activation and actin clustering at the cell leading edge. It has been proposed that ninjurin1 regulates cell migration via FAK signaling pathways. By showing the migration-inhibitory function of ninjurin1, we confirmed the importance of highly coordinated surface ninjurin1 and FAK signaling in regulating cell migration. Furthermore, enhanced cell migration and FAK phosphorylation were observed in siRNA-mediated ninjurin1 knock-down in the macrophage cell line RAW264.7. Clearly, ninjurin1 is a pivotal regulator of macrophage migration and our data show that ninjurin1 is a suppressor of the FAK-dependent cell migration.

In conclusion, we demonstrate that ninjurin1 can inhibit macrophage migration via suppression of FAK signaling. FAK is a critical player here since its down-regulation mediated by ninjurin1 overexpression results in decreased macrophage migration. Considering that the inhibition of tumor infiltration by macrophages contributes to the suppression of tumor growth and tumor angiogenesis, our data highlight the potential of ninjurin1 as a therapeutic target. By regulating ninjurin1 expression, we might control inflammation mediated tumors.

## MATERIALS AND METHODS

### Colitis-associated colon cancer model (CAC)

Ninjurin1 transgenic mice (ninjurin1 tg mice) harboring a LoxP-Stop-LoxP-ninjurin1 construction were activated by Cre-mediated excision of the floxed-Stop sequence [32]. Ninjurin1 tg mice were generated using pronuclear injection of purified LoxP-Stop-LoxP-ninjurin1 construction. Transgenic mice were identified by PCR and bred with *Lysozyme 2 –Cre knock in mice (B6.129P2-Lyz2*^tm1(cre)Ifo^ */J) [purchased from Jackson Laboratory (Bar Harbor, ME, USA)]*. Double transgenic mice were backcrossed to FVB six times (Figure 1A). The colitis-associated colon cancer (CAC) model was developed, as described previously [33]. Briefly, wild type mice (wt mice) and ninjurin1 tg mice were injected intraperitoneally with 10 mg/kg of AOM (Sigma, St Louis, MO, USA) in phosphate-buffered saline (PBS). One week later, 2% DSS (MP Biomedicals, Solon, OH, USA) was given in the drinking water for 7 days (Figure 1B). This DSS treatment was repeated twice again and finally followed by regular water until the end of the experiment. The mice were killed 14 weeks after injection of AOM. For macroscopic and histological examination of tumors, the colons were isolated, rinsed with PBS and then opened longitudinally. Tumors were counted and the size of each tumor was determined using a dissection microscope. All animal care and experimental procedures were approved by Institutional Animal Care and Use Committee (IACUC) at Gachon University in Incheon, Korea.

### Macrophage adoptive transfer

For adoptive transfer studies, 6 week-old female NOD/SCID mice (NOD/LtSz-Prkdcscid/J) were used to establish a tumor xenograft model. Mice were purchased from KRIBB (Korea Research Institute of Bioscience & Biotechnology, Cheongwon-gun, Korea). 1 × 10^7^ A549 cancer cells were subcutaneously inoculated in the right flank of each mouse. Mice were randomly divided into two groups: wt mice and ninjurin1 tg mice. When tumors reached volumes of approximately 300 mm^3^, fluorescence labeled macrophages were transferred into mice. Peritoneal resident macrophages were isolated from peritoneum of wt, ninjurin1 tg mice and ninjurin1 knock out mouse as described previously [34]. Ninjurin1 knock-out mice (provided by Prof. GT Oh) were used in this experiment [35, 36]. Cells were labeled with carboxyfluorescein succinimidyl ester (CFSE) as described previously [37]. The percentage of macrophages was between 40–50% as determined by flow cytometer (FACScan, BD Bioscience, San Jose, CA, USA) defining the macrophage phenotype as F4/80 positive cells. 5 × 10^6^ CFSE labeled cells were intracardially injected into mice. Recipient mice were sacrificed and tumors were dissected 24 h after adoptive transfer. Half of the tumor nodules were processed for immunofluorescence analysis [38] and the other half were dissociated and infiltrations were analyzed by flow cytometry.

### Immunofluorescence analysis

To characterize macrophage infiltrations, de-paraffinized sections of colon from wt and ninjurin1 tg mice were incubated with Alexa Fluor 488 conjugated Rat anti-mouse F4/80 antibody (BioLegend, San Diego, CA, USA). The slides were mounted with Vectashield mounting medium (Vector Laboratories Inc., Burlingame, CA, USA). We used an intravascular perfusion of Teaxs-red conjugated tomato-lectin (*Lycopersicon esculentum* lectin, Vector Laboratories Inc.) to label all blood vessels in colitis-associated colon cancers [39]. For this purpose, under anaesthesia, the mice were intracardially injected with 200 μl of tomato-lectin (0.5 mg/mL). Tomato-lectin binds uniformly to the luminal surface of endothelial cells [40] and can be used to identify all blood vessels. After injection, the tissues were processed for subsequent analyses as described above. For immunocytochemistry, mouse peritoneal macrophages grown on cover slips were fixed in cold methanol at −20°C for 15 min. Cells were incubated overnight with focal adhesion kinase (FAK) (Cell signaling Technology, Inc., Danvers, MA, USA) and actin antibodies (Santa Cruz Biotechnology, Inc., Santa Cruz, CA, USA). Cells were washed 3 times in PBS containing 0.1% Triton X-100, and then incubated with Alexa fluor-488 conjugated goat anti-mouse IgG (Life Technologies, Carlsbad, CA, USA) and PE conjugated rabbit anti goat IgG (Santa Cruz Biotechnology, Inc.) each for 2 h. The slides were mounted with Vectashield mounting medium.

### Flow cytometry analysis

For flow cytometry experiments, peritoneal macrophages were prepared from wt mice and ninjurin1 tg mice as described above, and splenic mononuclear cells were isolated as described below [41]. Spleens were harvested from wt mice and ninjurin1 tg mice, and minced by a pair of slide glass. After dissociation, splenocytes were forced and crushed through a cell strainer (SPL Life Sciences, Pocheon, Korea). Splenocytes were subjected to Ficoll-Hypaque (GE Health Care, Pittsburgh, PA, USA) density gradient centrifugation to isolate splenic mononuclear cells. These cells were then stained with sheep anti-ninjurin1 (R&D Systems, Minneapolis, MN, USA) and Alexa Fluor 488 conjugated Rat anti-mouse F4/80 antibody.

### Migration and invasion analysis

Cell migration and invasion assays were conducted using the Boyden chamber as described previously [42, 43]. The migration assay for cell migration was conducted using transwell ((8 μm pore size) BD Biosciences, Franklin Lakes, NJ, USA). In brief, cells were resuspended in serum-free medium, and 5 × 10^4^ cells/well were placed in the upper chamber of the transwell insert and 90% NIH-3T3 conditioned medium containing 10% FBS was added to the lower chamber and incubated for 8 h. For the migration assay, filters were coated with 0.1 mg/mL of collagen type IV (Trevigen, Gaithersburg, MD, USA) in PBS. The invasion assay was performed in the same manner except the transwell units were coated with matrigel (Becton Dickinson Labware, Bedford, MA, USA). Cells on the membranes were fixed and stained with hematoxylin. The numbers of cells in four independent fields were counted under a light microscope (Leica, Wetzlar, Germany). The results are expressed as the percentage of cells that migrated or invaded compared to control cells.

### siRNA knockdown

To knockdown ninjurin1, RAW264.7 cells were transfected with either siRNA directed against ninjurin1 (5ʹ- CCC ACU UUU CUA AUC AUG A -3ʹ) or nontargeting control siRNA (100 pmol/ml) using Lipofectamine RNAi MAX (Invitrogen/Life Technologies, Carlsbad, CA, USA) according to the manufacturer's instructions.

### Semi-quantitative RT-PCR and primers

For semi-quantitative RT-PCR, 1 μg of RNA was used as a template for reverse-transcription using the Prime Script 1'st strand cDNA Synthesis kit (Takara; Kyoto, Japan). PCR was carried out with cDNA using a PCR pre-mixture (Takara). The sequences of the primers used were as follows: *Actin* (349 bp; forward: 5ʹ-TGG AAT CCT GTG GCA TCC ATG AAA C-3ʹ; reverse: 5ʹ-TAA AAC GCA GCT CAG TAA CAG TCC G-3ʹ), *ninjurin1* (110 bp; forward: 5ʹ-TCA TCG TCG TGG TCA ACA TCT TC-3ʹ; reverse: 5ʹ-GCA GGT CCG GTA CCC TTA AAG TC-3ʹ), *TNF-*α (258 bp; forward: 5ʹ-ATA GCT CCC AGA AAA GCA AGC-3ʹ; reverse: 5ʹ-CAC CCC GAA GTT CAG TAG ACA-3ʹ), *IL-1*β (178 bp; forward: 5ʹ-ACC TGC TGG TGT GTG ACG TT-3ʹ; reverse: 5ʹ-TCG TTG CTT GGT TCT CCT TG-3ʹ), *IL-6* (155 bp; forward: 5ʹ-TGG AGT CAC AGA AGG AGT GGC TAA G -3ʹ; reverse: 5ʹ- TCT GAC CAC AGT GAG GAA TGT CCA C -3ʹ), *IL-12p40* (214 bp; forward: 5ʹ-GTC CTC AGA AGC TAA CCA TC -3ʹ; reverse: 5ʹ-TTT CCA GAG CCT ATG ACT CC-3ʹ), *iNOS* (487 bp; forward: 5ʹ-GTG GTG ACA AGC ACA TTT GG-3ʹ; reverse: 5ʹ-GGC TGG ACT TTT CAC TCT GC-3ʹ), *IL-10* (216 bp; forward: 5ʹ-ATG CTG CCT GCT CTT ACT GAC TG-3ʹ; reverse: 5ʹ-CCC AAG TAA CCC TTA AAG TCC TGC-3ʹ), *TGF-*β (298 bp; forward: 5ʹ-CTT CAG CTC CAC AGA GAA GA-3ʹ; reverse: 5ʹ- CAC GAT CAT GTT GGA CAA CTG-3ʹ), *VEGF*(716 bp, 644 bp, and 512 bp; forward: 5ʹ-GCG GGC TGC CTC GCA GT -3ʹ; reverse: 5ʹ-TCA CCG CCT TGG CTT GTC A -3ʹ), *PDGF* (644 bp; forward: 5ʹ-AGC ATC CGG GAC CTC CAG -3ʹ; reverse: 5ʹ-AAG ACC GCA CGC ACA TTG G-3ʹ), and *FGF2* (328 bp; forward: 5ʹ-ACA CGT CAA ACT ACA ACT CCA-3ʹ; reverse: 5ʹ-TCA GCT CTT AGC AGA CAT TGG -3ʹ).

### Western blotting

Western blot analysis was performed, as described previously [44]. Briefly, whole-cell lysates were prepared in a modified RIPA buffer containing proteinase inhibitors and phosphatase inhibitors as described elsewhere [45]. Equivalent amounts of protein (20–80 μg) were loaded in 10% or 12% sodium dodecyl sulfate–polyacrylamide gel electrophoresis (SDS–PAGE) gels and transferred by blotting to polyvinylidene fluoride (PVDF) membranes. The blot was incubated with primary antibodies against pFAK (Tyr 925; Cell Signaling Technology), pFAK (Tyr 576/577: Cell signaling technology), total FAK, pAKT, AKT, pERK1/2, ERK1/2 (Cell Signaling Technology), and actin (Santa Cruz Biotechnology, Inc.). After washing, the blot was incubated with HRP-conjugated secondary antibodies. The protein–antibody complexes were detected using enhanced chemiluminescence (Amersham, Arlington Heights, IL, USA), according to the manufacturer's recommended protocol.

Western blot bands were quantified using Image J software (NIH, Bethesda, MD, USA). The adjusted relative densities were calculated relative to the expression of control sample's actin

### Statistical analysis

All experiments were done in duplicate or triplicate. A two-tailed Student's *t*-test was used for statistical analysis of comparative data using Microsoft Excel software (Microsoft Co., Redmond, WA, USA). Values of *p* < 0.05 were considered significant and indicated by asterisks in the figures. For graphical representation of data, y-axis error bars indicate the standard deviation (s.d.) of the data for each point marked on the graph.

## SUPPLEMENTARY MATERIALS FIGURES AND TABLES


