# Supplementary material for: Ninjurin1 inhibits colitis-mediated colon cancer development and growth by suppression of macrophage infiltration through repression of FAK signaling
Source: Oncotarget. 2016 Apr 26;7(20):29592–604. doi: 10.18632/oncotarget.9020 (PMC5045419; doi:10.18632/oncotarget.9020)
Supplement: Supplementary file 1 [file oncotarget-07-29592-s001.pdf]

Ninjurin1 inhibits colitis-mediated colon cancer development and growth by suppression of macrophage infiltration through repression of FAK signaling

Supplementary Materials

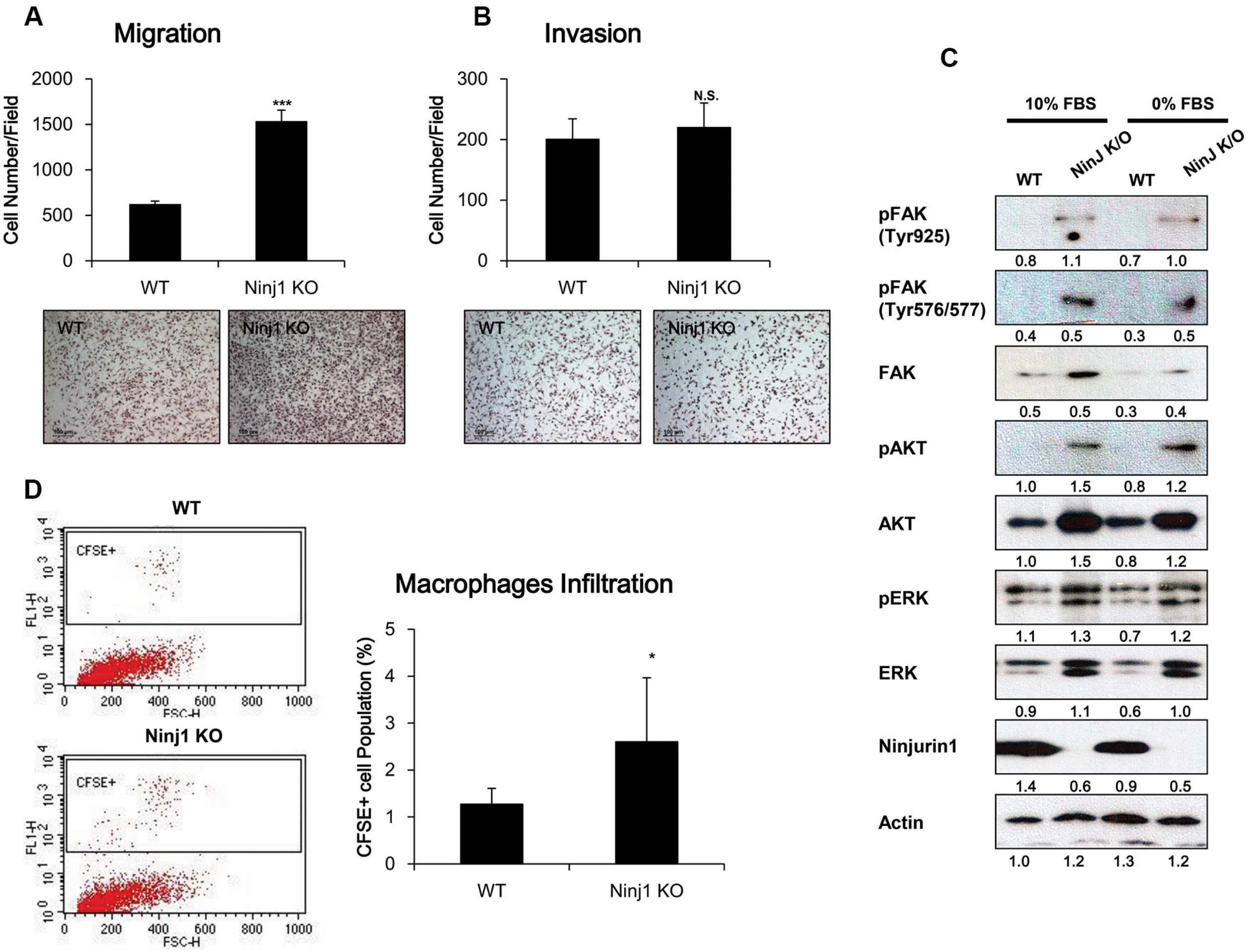

Supplementary Figure S1: Ninjurin1 knock-out ~~on~~ in macrophage induces cell migration and tumor infiltration. Ninjurin1 knock-out macrophages migration (**A**) and invasion (**B**). Migrated and invasive macrophages on outer surface of the upper chambers were stained with H&E. (**C**) Expression of FAK, phosphorylated FAK, phosphorylated AKT and phosphorylated ERK in ninjurin1 knock-out macrophages were detected by western blotting. (**D**) CFSE labeled macrophages were observed in xenograft tumors collected from recipient mice. Differences were evaluated using an unpaired two-tailed Student's *t*-test. (Error bars denote the standard deviation [SD]) (*$p < 0.05$ and ***$p < 0.001$).
